# Characterizing human decellularized crystalline lens capsules as a scaffold for corneal endothelial tissue engineering

**DOI:** 10.1002/term.2633

**Published:** 2018-02-11

**Authors:** Bert Van den Bogerd, Sorcha Ní Dhubhghaill, Nadia Zakaria

**Affiliations:** ^1^ Ophthalmology, Visual Optics and Visual Rehabilitation, Translational Neurosciences, Faculty of Medicine University of Antwerp Wilrijk Belgium; ^2^ Department of Ophthalmology Antwerp University Hospital Edegem Belgium; ^3^ Centre for Cell Therapy and Regenerative Medicine Antwerp University Hospital Edegem Belgium

**Keywords:** biological scaffold, cornea, corneal endothelial cells, lens capsule, regenerative medicine, tissue engineering

## Abstract

The idea of transplanting a sheet of laboratory‐grown corneal endothelium dates back to 1978; however, the ideal scaffold is still lacking. We hypothesized that human crystalline lens capsules (LCs) could qualify as a scaffold and aimed to characterize the properties of this material for endothelial tissue engineering. LCs were isolated from donor eyes, stored at −80 °C, and decellularized with water and trypsin‐EDTA. The decellularization was investigated by nuclear staining and counting and the capsule thickness was determined by optical coherence tomography and compared with Descemet's membrane (DM). Transparency was examined by spectrometry, and collagenase degradation was performed to evaluate its resistance to degradation. Cell‐scaffold interaction was assessed by measuring focal adhesions surface area on LC and plastic. Finally, primary corneal endothelial cells were grown on LCs to validate the phenotype. Trypsin‐EDTA decellularized most effectively, removing 99% of cells. The mean LC thickness was 35.76 ± 0.43 μm, whereas DM measured 25.93 ± 0.26 μm (p < .0001). Light transmission was 90% for both LC and DM. On a collagenase challenge, LC and amniotic membrane were digested after 13 hr, whereas DM was digested after 17 hr. The surface area of focal adhesions for cells grown on coated LCs was at least double that compared with other conditions, whereas tight junctions, ion pumps, and hexagonal morphology were well maintained when endothelial cells were cultured on LCs. In conclusion, LCs demonstrate excellent scaffolding properties for tissue engineering and sustain the cell phenotype and can be considered a suitable substrate for ocular tissue engineering or as a template for future scaffolds.

## 1 INTRODUCTION

Corneal opacities account for 5% of blindness worldwide and have been listed as the fourth leading cause of blindness globally (WHO, 2015). Corneal pathologies, including hereditary diseases, ocular burns, and trauma to the eye, often render the cornea irreversibly opaque in the long term. In most cases, corneal clarity can only be re‐established by replacing it with a donated cadaveric donor cornea. In 2015, U.S. eye bank reports stated that 40% of all corneal transplantations were performed for pathologies of the corneal endothelium (Eye Bank Association of America, 2016). The endothelium covers the inner surface of the cornea and is crucial for its transparency. On a cellular level, it is arranged as a monolayer of cells which maintains the optimal corneal thickness by a “pump‐and‐leak mechanism.” When this balance is disturbed, water swells into the corneal lamellae, the crucial collagen alignment in the cornea is lost, and the cornea becomes cloudy.

Corneal endothelial cells are growth‐arrested and not thought to be capable of regeneration. Over a lifetime, nonviable cells are shed and replaced by enlargement and migration of adjacent endothelial cells. This results in an age‐related decline in endothelial cell density accompanied by an increase in cell size. If the cell density drops under a certain threshold (approximately 500 cells/mm^2^), the pump and leak function fails and corneal oedema ensues (Peh, Beuerman, Colman, Tan, & Mehta, 2011). Endothelial keratoplasty (EK) is the current surgical standard of care for clinically significant endothelial failure. During EK, the dysfunctional endothelial cell layer is removed and replaced by donor endothelium on Descemet membrane (DMEK) or Descemet membrane with corneal stroma (DSAEK). Unfortunately, a global scarcity in corneal donors limits treatment and has resulted in long waiting times for transplant recipients (Gain, Jullienne, & He, 2015).

Various experimental approaches are being investigated to increase the number of patients that can be treated by a single donor cornea by ex vivo expansion of primary corneal endothelial cells. The main challenges of this approach lie not only the difficulty of expanding corneal endothelial cells but also designing a method of delivering the cells to the eye. The two delivery systems have been described, namely, (a) an intracameral injectable endothelial cell suspension, or (b) tissue‐engineered endothelial grafts utilizing a carrier membrane or scaffold. Culturing the cells on a scaffold resembles the current endothelial surgical techniques, and as such, the majority of research groups involved in this domain are investigating the scaffold‐based approach (Navaratnam, Utheim, Rajasekhar, & Shahdadfar, 2015).

A cell scaffold provides mechanical support to a graft during transplantation; however, it can also influence fundamental cellular processes such as proliferation and differentiation through its topography and elasticity (Chan & Leong, 2008; Palchesko, Lathrop, Funderburgh, & Feinberg, 2015). When used in corneal endothelial tissue engineering, the scaffold must also be transparent and thin enough to avoid aberrations when transplanted in patients' visual axis. Corneal scaffolds must also be avascular and permeable to the aqueous, which supplies nutrients to the posterior corneal stroma. Lastly, the scaffold should also be cytocompatible and favour cellular adhesion and proliferation (Navaratnam et al., 2015).

The human crystalline lens capsule (LC) is an epithelial basement membrane that completely encapsulates the crystalline lens and forms the anatomical separation of the anterior and posterior segment of the eye. The LC is composed of collagen IV and laminin and structurally divided at the equatorial region into an anterior and posterior capsule. The lens epithelial cells deposit extracellular matrix onto the capsule, and thus, the LC gradually thickens with age (Danysh & Duncan, 2009). The capsule is inherently transparent and can form a roll when submersed in water, behaving similar to the Descemet membrane making it an attractive scaffold option (Droutsas et al., 2014). Anterior capsular material could potentially be obtained as a by‐product of cataract surgery, one of the most frequently performed surgeries in the world. Preliminary studies have investigated the growth of corneal derived cells on the LC, namely, corneal epithelial and endothelial cells (Albert et al., 2012; Kopsachilis, Tsinopoulos, Tourtas, Kruse, & Luessen, 2012; Yoeruek et al., 2009). These studies however did not examine the additional physical properties of the LC that could make it suitable as a corneal endothelial scaffold.

In this study, we investigated the decellularization process, thickness, transparency, cell‐scaffold adhesion, and biodegradation of the LC and compared it with Descemet's membrane (DM) and human amniotic membrane (HAM), widely used in ocular surgery. As a final proof of concept, primary corneal endothelial cells were seeded on these substrates and investigated immunohistochemically for phenotype and morphology.

## 2 MATERIAL AND METHODS

### 2.1 Isolation and decellularization of HAM‐DM‐LC

An ethical approval was set in place for use of donor eyes in research (EC14/30/319). HAMs (*n* = 3) were prepared as previously described and cryopreserved at −80 °C (Zakaria et al., 2010). After thawing, the membrane was treated with thermolysin (Invitrogen, California, USA), to remove the amniotic epithelium, and the spongy layer was manually removed by hydration followed by scraping. DM (*n* = 2) was obtained from research‐grade donor eyes. Briefly, after isolation of the cornea, a circular cut was made on the inner aspect of the trabecular meshwork with a D800 Muraine punch (Moria, Antony, France). The DM was peeled off with fine forceps and stored at 4 °C in sterile phosphate buffered saline solution (Life Technologies, California, USA). LCs were dissected from research grade donor eyes by scoring a cross in the posterior capsule removing the lens material. Anterior LCs were mounted on nitrocellulose membranes and stored at −80 °C in Coldstore medium (Eurobio, Les Ulis, France). To validate the decellularization of LCs, thawed and fresh specimens were washed in sterile H_2_O (Baxter, Deerfield, USA) or Trypsin‐EDTA (Life Technologies, California, USA) twice for 5 min to remove remaining lens epithelial cells. Samples (*n* = 12) were then fixed in 4% paraformaldehyde (PFA) and permeabilized with Triton X‐100 for 30 min. The nuclei were stained with DAPI (1:100, Sigma Aldrich, St. Louis, USA). Every test condition was performed in duplicate, and three representative pictures were taken (Nikon Ti‐E). LCs that were freshly dissected or frozen, but not subsequently treated, served as negative controls.

### 2.2 LC thickness

LC (*n* = 3) and DM (*n* = 3) thickness was measured using an optical coherence tomography (OCT) apparatus (OptoVue iVue OCT). Samples were measured, without being fixated, at room temperature and were kept hydrated during measurements. All measurements were performed on glass slides, and 10 images were taken per sample. Fifteen measurements were performed on every image using imageJ software, with the images being calibrated using the internal scale of the iVue software.

### 2.3 Light transmission of the LC

Light transmission values were obtained by measuring absorbance at specific wavelengths and converting them to light transmittance (Duan & Sheardown, 2006; Rafat et al., 2016). LC (*n* = 3), DM (*n* = 2), and HAM (n = 3) were trephined to fit the 6‐mm diameter of 96‐well ViewPlates (Perkin Elmer, Massachusetts, USA). Light absorbance was measured at room temperature for specific wavelengths within the visual spectrum (405, 450, 490, 530, and 630 nm) with the Wallac 1420 VICTOR^3^ microplate reader (Perkin Elmer, Massachusetts, USA). Measurements were repeated three times. Absorbance values were then converted to the percentage of light transmittance with the following equation (assuming that there is little to no reflection):

$$A=2-\log\left(\%T\right)$$

$$\to \%T=10^{\left(2-A\right)}$$

where A is absorbance and %T is the percentage of transmittance. In this experiment, Trypan Blue (Life Technologies, California, USA) and demineralized water served as negative and positive controls, respectively. Light transmittance was compared with the HAM and the DM. Transmittance values were normalized to demineralized water as a positive control.

### 2.4 Biodegradation of the LC

Biodegradation was measured in vitro as previously reported (Rafat et al., 2016). Collagenase type IA (Sigma, St. Louis, USA) was diluted in Tris–HCl buffer (0.1 M, pH 7.4; VWR, Radnor, USA) to a 5 U/mL solution. Surface water was blotted away, and samples were weighed with a Sartorius SE2 ultramicrobalance (Sartorius, Göttingen, Germany) at different time points (0–780 min), whereas collagenase solution was refreshed every 8 hr. LCs (*n* = 4), DM (*n* = 3), and HAM (*n* = 3) samples were prepared as previously described, and all samples had a consistent diameter of approximately 10 mm. As controls, LC (*n* = 3) and HAM (n = 3) were incubated in Tris–HCl buffer only and weighed at the same time points. Residual mass was calculated with the following equation: Residual mass (%) = W_t_/W_0_%, where W_t_ represents tissue weight at particular time points and W_0_ is the initial weight. At each time point, each LC and HAM sample was measured three times independently.

### 2.5 Cell‐scaffold interaction

Cellular attachment on different scaffolds was quantified by immunohistochemical staining of focal adhesions, which are accumulations of integrins that link the cytoskeleton to the extracellular matrix. The experimental conditions consisted of LCs and tissue culture plastic as a control, both uncoated and coated with FNC coating mix (Athena Enzyme Systems, Baltimore, USA), and were each seeded with approximately five thousand cells. Forty‐eight hours later, the samples were fixed in 4% PFA and permeabilized with Triton X‐100. The primary anti‐Vinculin antibody (1:200, Abcam, Cambridge, UK) was incubated overnight, and secondary antibody GAM‐FITC (1:100, Jackson Immunoresearch, West Grove, USA) was incubated for two additional hours. Cell nuclei were counterstained with DAPI (Sigma, St. Louis, USA). Samples were imaged using with a Nikon Ti‐E confocal microscope (Nikon, Tokyo, Japan) and processed with imageJ with an in‐house developed macro for automatic processing of the surface area of focal adhesions.

### 2.6 Primary cells grown on LCs

Primary human corneal endothelial cells (HCEnCs) were isolated as previously reported, from research grade donor eyes (Peh et al., 2013). In short, corneas (*n* = 7; aged 63, 70, 75, and 80 years old) were inversely mounted on a suction cup under a dissecting microscope where the endothelial layer was gently peeled off using forceps. Trypan blue counterstain was used to ensure complete removal of the DM and corneal endothelial cells. Cells were dissociated from the basement membrane using a Collagenase 1A digest (Sigma, St. Louis, USA) for 2 hr at 37 °C, 5% CO_2_ and placed on cell shaker (Biosan, Riga, Latvia). Once the cells had detached, a secondary digest with TrypLE Express was performed for 5 min (37 °C) to obtain a single cell suspension. Both corneas from the same donor (*n* = 3) were pooled and seeded on two LCs and in two control wells (tissue culture plastic) in a 24‐well plate. All four wells were coated with FNC coating, and the cells grew to confluency within 2 weeks. Silicone rings (Rubbermagazijn.nl, Zoetermeer, The Netherlands) were mounted in all conditions to obtain an equal surface area for cells to grown on (inner diameter = 9 mm). After reaching confluency, HCEnCs were fixed in 4% PFA and permeabilized with Triton X‐100. Samples were incubated overnight with a primary anti‐ZO‐1 (1:200, Thermo Fisher Scientific, Massachusetts, USA) or anti‐Na^+^/K^+^ATPase antibody (1:40, Santa Cruz Biotechnology, Texas, USA). The secondary antibody DAM‐Cy3 (1:100, Jackson Immunoresearch, West Grove, USA) was incubated for 2 hr, and nuclei were counterstained using DAPI (Sigma, St. Louis, USA).

### 2.7 Data analysis

Data are expressed as mean ± SEM. Statistical analysis was performed using GraphPad Prism 6.0. After data exploration, data were tested for normality using the sample size, Kolmogorov–Smirnov test, and histograms. Parametric testing was performed to check for a significant difference between test conditions; otherwise, nonparametric tests were used.

## 3 RESULTS

### 3.1 Decellularization of human anterior LCs

**Figure 1:**
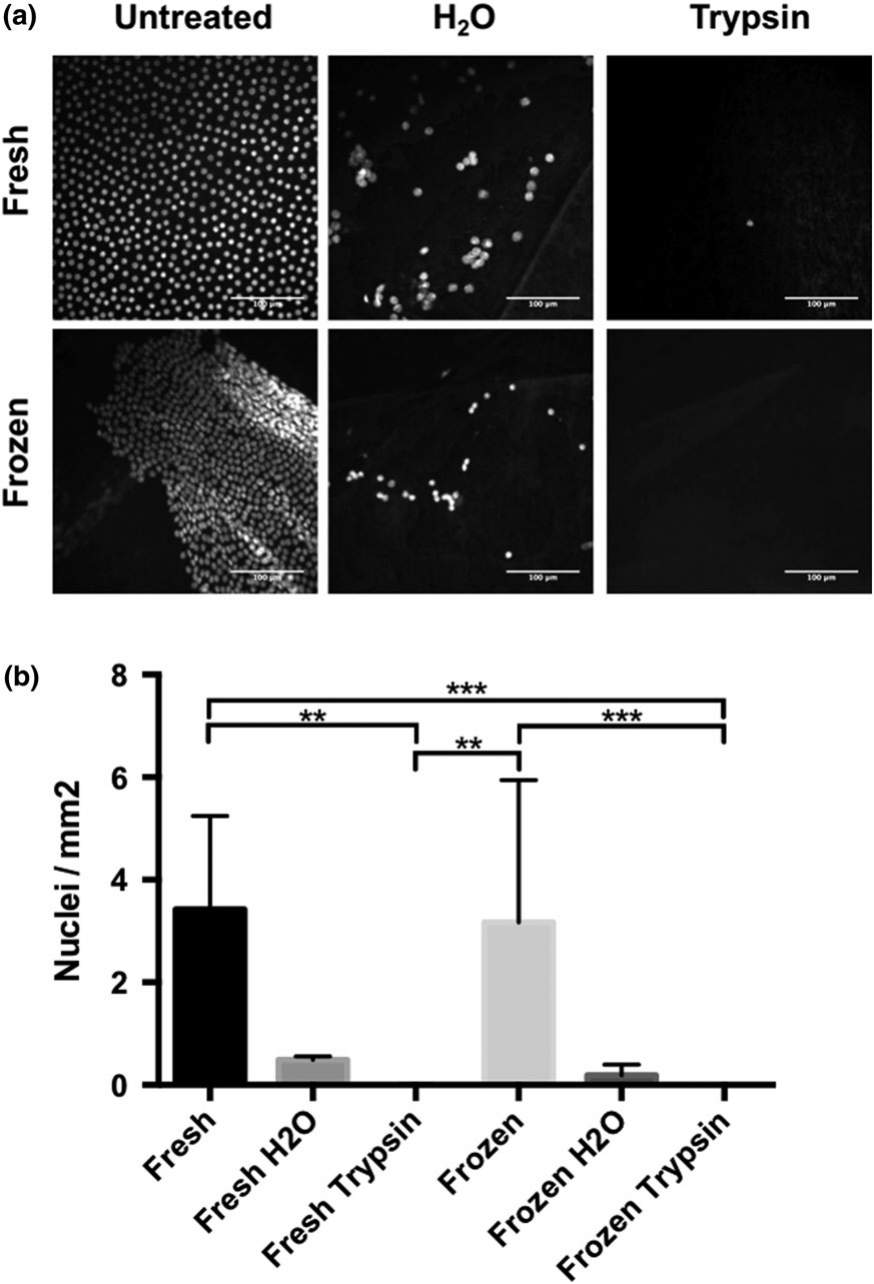
Decellularization of the lens capsule (LC). (a) Remaining nuclei were detected with DAPI nuclear staining and (b) automatically counted. Frozen LC samples that were treated with Trypsin‐EDTA were completely debrided of cells (**p < .05, ***p < .001)

Human LCs were decellularized either freshly dissected or following a freeze thaw cycle at −80 °C. DAPI staining was performed to quantify the remaining cells and expressed as the amount of nuclei/mm^2^ of LC (Figure 1). Untreated control samples were seen to be populated with lens epithelial cells, whereas all frozen specimens showed partial cell detachment (Figure 1a, left panel). Even after washing in sterile H_2_O, some cells remained on both the frozen and fresh samples. Rinsing with trypsin‐EDTA was most effective in both fresh and frozen groups, removing almost 100% of all the lens epithelial cells. Based on these results, every subsequent LC was de‐epithelialized using the optimized 2 × 5 min trypsin‐EDTA protocol.

### 3.2 LC dimensions

**Figure 2:**
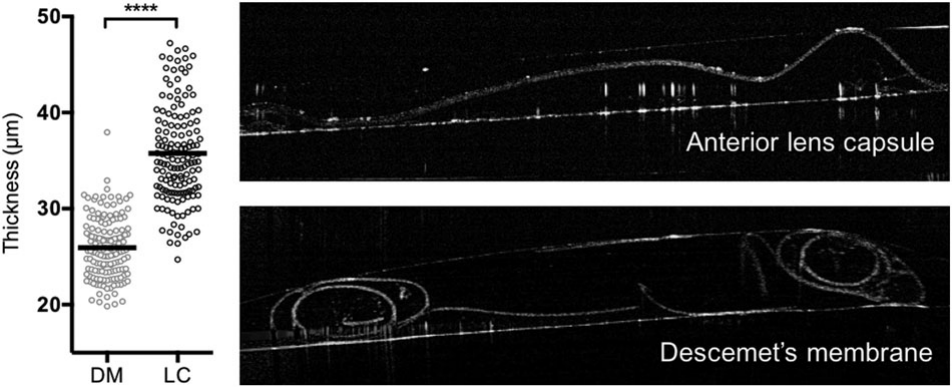
Thickness of the lens capsule (LC) and Descemet's membrane (DM) with a black bar indicating the mean thickness value. Optical coherence tomography images of both membranes are displayed on the right side (****p < .0001)

After isolation, LC samples measured approximately 10 mm in diameter. LC thickness was in average 35.76 ± 0.43 μm compared with the thinner DM of 25.93 ± 0.26 μm (Mann–Whitney test, *p* < .0001; Figure 2).

### 3.3 Transparency of the LC

**Figure 3:**
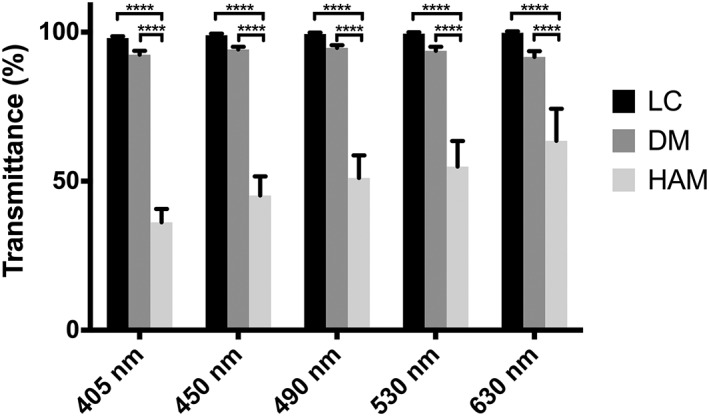
Transparency values of the lens capsule (LC), Descemet's membrane (DM), and human amniotic membrane (HAM). Both LC and DM were almost completely transparent. The HAM, on the contrary, was around 50% transparent (****p < .0001)

The LC displayed highest values for light transmission, which were not significantly lower than the positive controls: an empty well and H_2_O (two‐way analysis of variance, *p* = .8662; Figure 3). Transmission of light was higher than 98% and 90% for the LC and DM, respectively. There was no significant difference between the LC and DM in the visual range. On the other hand, HAM was significantly less transparent at each wavelength compared with DM and LC (two‐way analysis of variance, *p* = .0003).

### 3.4 Biodegradation of the LC

**Figure 4:**
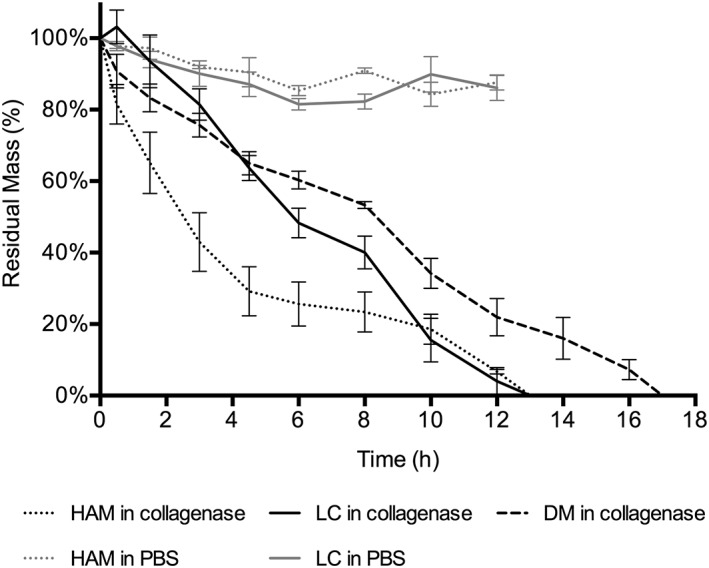
During the collagenase challenge test, the Descemet's membrane (DM) took the longest to degrade, whereas the human amniotic membrane (HAM) and lens capsule (LC) were completely digested after 13 hr. The negative controls were incubated in phosphate buffered saline (PBS) and did not degrade over time

Figure 4 shows the results of the collagenase challenge test performed to compare the degradation of the human LC with the HAM and DM. HAM showed a steep degradation curve initially, but both the LC and HAM were completely digested after 13 hr. The DM was completely digested after 17 hr. HAM degraded by 50% in 2–3 hr, whereas it took 6 hr for the LC and 8 hr for the DM to attain 50% digestion.

### 3.5 Cell‐scaffold interaction

**Figure 5:**
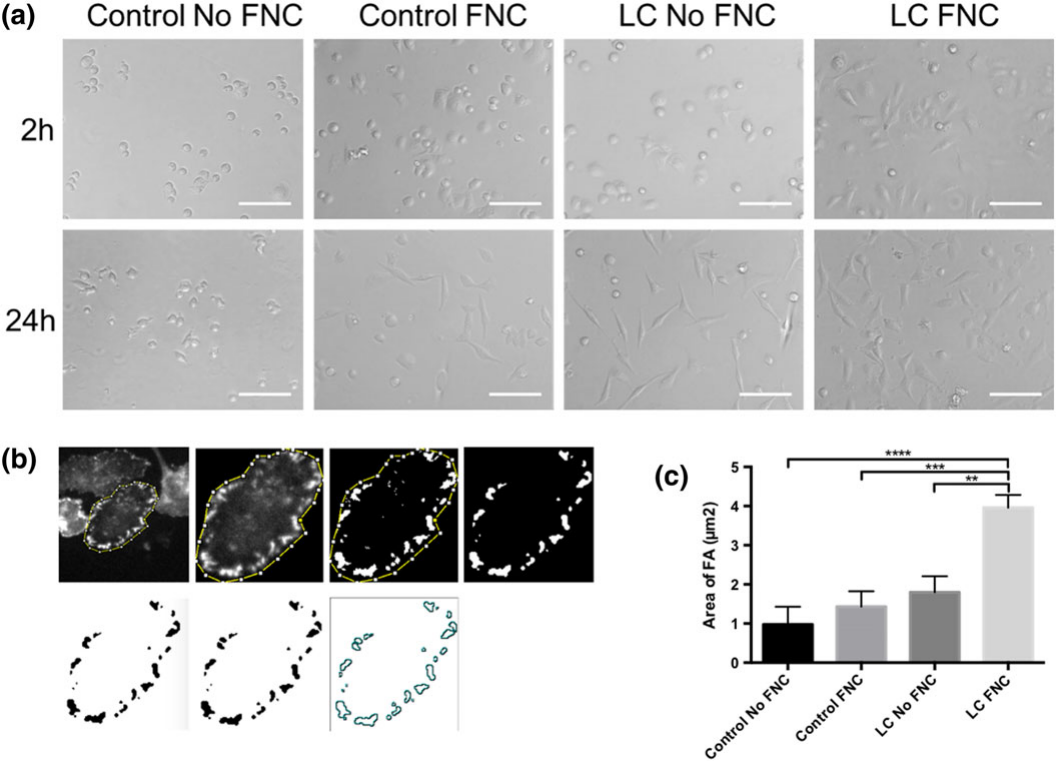
Cellular attachment to the lens capsule (LC). (a) After 2 hr, cell spreading was noticed on coated FNC the most. Twenty‐four hours later, every condition displayed properly attached cells except for uncoated plastic. (b) Cells were stained for vinculin and processed accordingly. (c) The average surface area of focal adhesions for each cell was calculated and was seen to be the highest on coated LC and lowest on uncoated plastic (**p < .05, ***p < .001, ****p < .0001)

After 2 hr, cell spreading indicating cellular attachment was most prominent on the LC coated with FNC and to a lesser degree coated plastic. When observed 24 hr later with bright field microscopy, cells were spread evenly in every condition, except for the uncoated plastic, which showed only minimal cell attachment (Figure 5a). Forty‐eight hours after seeding, the calculated surface area of focal adhesions was significantly higher on coated LC than on other substrates (Kruskal‐Wallis test, *p* < .05; Figure 5a,b).

### 3.6 Primary cells on LC phenotype.

**Figure 6:**
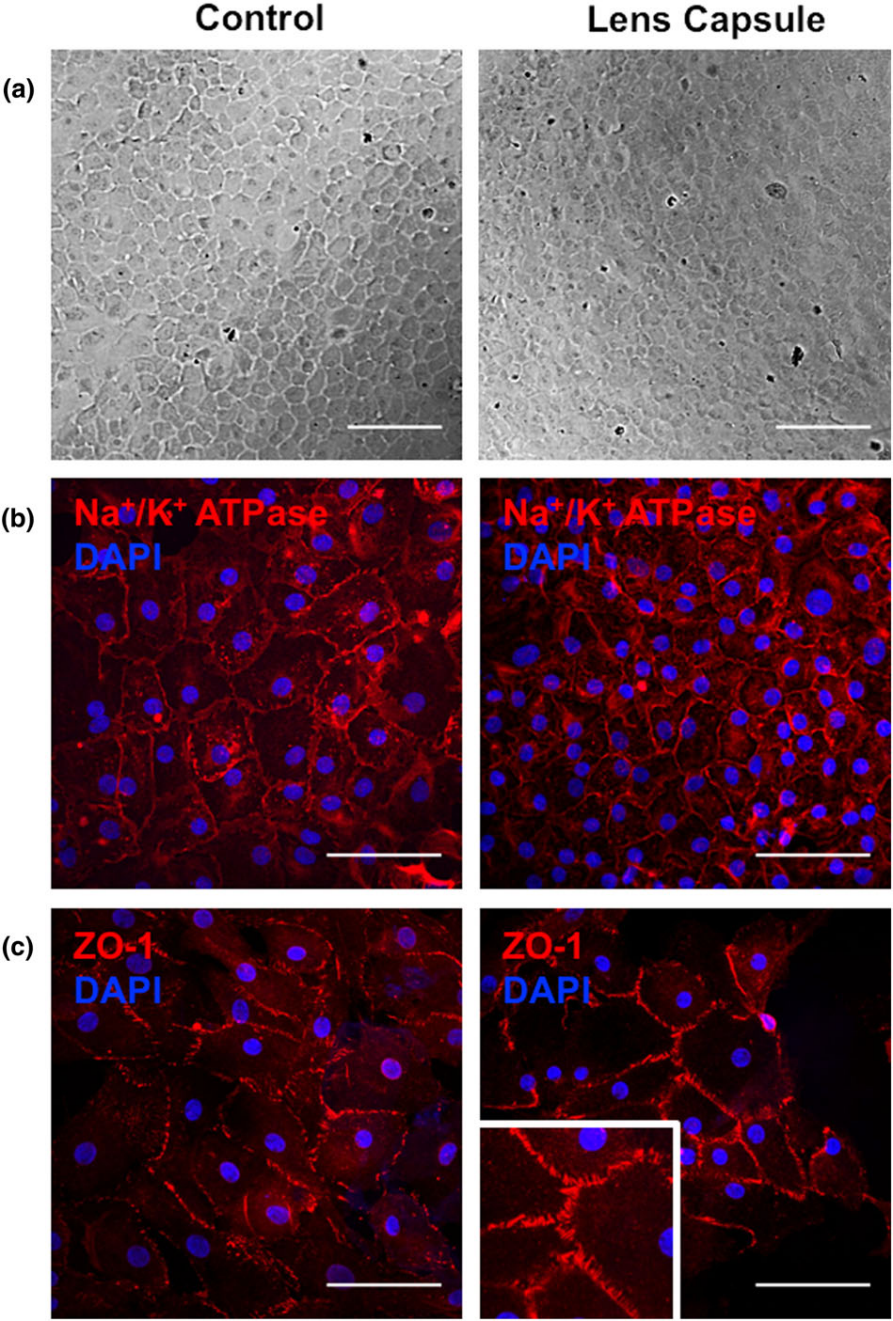
Primary human corneal endothelial cell grown on lens capsule (LC) showed a similar hexagonal appearance (a) on plastic and on the LC (scale bar = 200 μm). (b) Similarly, the qualitative Na^+^/K^+^ ATPase and ZO‐1 expression, representing their pumping and leaking capacity respectively, were also comparable (scale bar = 100 μm)

Primary corneal endothelial cells grown on LC showed a comparable hexagonal morphology as with those grown on plastic (Figure 6a). Na^+^/K^+^ ATPase ion pumps were present in the same degree on the basolateral membrane of cells cultured in both conditions (Figure 6b). Similarly, ZO‐1 appeared to be present as a cortical band of tight‐junctions all throughout the formed monolayer grown on plastic and LC (Figure 6c).

## 4 DISCUSSION

The use of LC for ocular repair has previously been investigated in corneal epithelial diseases. Capsules containing their intact epithelial cells have been used in a rabbit model of mechanical injury (Feng, Borrelli, Reichl, Schrader, & Geerling, 2014; Kozak, Trbolova, Kolodzieyski, Juhas, & Ledecky, 2003). Transplanting the LC with the epithelial side down resulted in a more rapid recovery and a transparent cornea at 6 months when compared with control eyes. LC has also been used successfully as a scaffold for the expansion of both limbal epithelial stem cells and HCEnC in vitro (Kopsachilis et al., 2012; Lachaud et al., 2014; Navaratnam et al., 2015; Yoeruek et al., 2009). These data suggested that LC could eventually be used for human transplantation, but prior to in vivo use, further characterization is required. In these experiments, we optimized the decellularization process and characterized the membrane for its thickness, transparency, rate of biodegradation, cell‐scaffold interaction, and its biocompatability with primary HCEnC.

Anterior LC thickness has been reported in the literature in the range of 8–27 μm using electron microscopy, light microscopy, and microspherules (Danysh, Czymmek, Olurin, Sivak, & Duncan, 2008). We used OCT as a noncontact, cross‐section imaging technique to perform our measurements ex vivo as it is widely used for both qualitative and quantitative measurements of the anterior segment of the eye (Han, Liu, Noriega, & Mehta, 2016). Our mean measured thickness of 35 μm is higher compared with the previous reported values. We believe this could be due to the resolution of the OCT readout that is limited to 5 μm and the absence of tissue fixation prior to measuring. When using an LC as a scaffold, the resulting composite cell graft would eventually be thicker than a DMEK graft of around 20 μm. However, it would still be thinner than a DSAEK, which is approximately 100 μm thick (Daoud et al., 2013). Posterior LCs, on the other hand, would be thinner than the anterior, but this material would be more difficult to obtain as it is not a by‐product of cataract surgery. Additionally, the mechanical strength of posterior capsules is reported to be three to seven times lower than its anterior equivalent and is directly correlated to the difference in thickness (Krag & Andreassen, 2003).

Although the LC is an inherently transparent membrane, we examined its transparency after storage and decellularization to ensure that it would not be altered during the process. The preparation steps did not change the transparency, so LC remains a viable candidate scaffold. Unsurprisingly, HAM scored poorly on the transparency measurements, consistent with its use in vivo where it is used to patch urgent corneal defects at the cost of inducing corneal haze (Deihim, Yazdanpanah, & Niknejad, 2016). During the degradation assay, HAM initially degraded at a faster rate, perhaps because it did not scroll like the DM and LC, exposing a larger surface area to the enzyme. Complete degradation of the HAM, however, occurred at the same time as the LC.

Cellular attachment to a substrate begins when cells first sediment onto the substrate and passively adhere by electrostatic interaction. Cells subsequently secrete extracellular matrix providing anchorage points for integrins to bind. Integrins cluster and form focal adhesion plaques that link the actin cytoskeleton to the matrix (Khalili & Ahmad, 2015). The fibronectin‐coated LC proved beneficial as the cells could immediately begin binding their integrins to the extracellular matrix molecules in contrast to plastic. The difference in the area of focal adhesions formed between the uncoated and coated LC is likely due to the increased availability of the R‐G‐D sequence. This sequence is the essential binding motif of integrins and is contained in the FNC mix. As the LC primarily consists of collagen IV and laminin, the fibronectin coating is able to provide the additional binding points for the cellular integrins (Danysh & Duncan, 2009; Takagi, 2004).

Currently, there is no consensus on a specific HCEnC marker, but during the past 5 years, numerous attempts have been made to identify proper HCEnC defining markers using gene expression profiling, immunocytochemistry, whole cell immunization, flow cytometry, transcriptomic analysis, and phage display (Cheong et al., 2013; Chng et al., 2013; Ding, Chin, Peh, Mehta, & Choo, 2014; Dorfmueller et al., 2016; Frausto, Le, & Aldave, 2016; Okumura et al., 2014; Yamaguchi et al., 2015; Yoshihara et al., 2015). Remarkably, CD166 was identified by three independent groups using different techniques (Ding et al., 2014; Dorfmueller et al., 2016; Okumura et al., 2014). However, up to this day, the presence of Na^+^/K^+^ ATPase and ZO‐1 and their hexagonal morphology most often serve as hallmarks of healthy corneal endothelial cells. These two markers represent the main functions of the corneal endothelium, namely, pumping (Na^+^/K^+^ ATPase) and barrier formation (ZO‐1), and were demonstrated as present on the LC endothelial cultures. It is important to validate the correct phenotype of primary cells when grown on different substrates as surface topography and elastic modulus can have an influence on this (Palchesko et al., 2015).

Although it was possible to characterize many parameters of LC for its use as an endothelial scaffold, there are still issues that require examination before application in humans. Controlled permeability is a key function of the endothelial barrier, but this was not modelled in our experiments. Previous reports already have demonstrated that the LC allows diffusion of small molecules such as glucose, O_2_, CO_2_, and waste products of lens epithelial cells, but this has yet to be investigated in LC endothelial cultures in vitro. (Danysh & Duncan, 2009; Friedenwald, 1930).

In these experiments, we used LCs derived from cadaveric research‐grade eyes. However, to shorten current transplantation waiting lists, the amount of available scaffolds should exceed the amount of corneal donors to eventually make multiple transplants from one donor cornea. LCs could potentially be sourced from cataract surgeries; however, we have not investigated whether the cataract influences the LC parameters. Also, these samples would have a limited size of 5–6 mm, whereas DMEK grafts are usually around 8–9 mm in diameter (Price, Gupta, Lass, & Price, 2017). However, an increasing amount of reports challenge that “perfect anatomical replacement,” represented by current EK techniques, is mandatory for corneal clearing (Van den Bogerd, Dhubhghaill, Koppen, Tassignon, & Zakaria, 2017). Hemi‐DMEK, for instance, is a proposed technique that uses only half a DMEK graft to transplant, leaving behind areas of uncovered stroma, but shows similar outcomes to traditional EK up to 3 years later (Lam et al., 2014; Lam et al., 2015; Müller, Baydoun, & Melles, 2016). Baring this in mind, smaller grafts could prove to be more effective than traditional grafts that completely cover the dissected posterior cornea.

## 5 CONCLUSION

This work establishes features of the LC that make it suitable as a scaffold for corneal tissue engineering and provides a comparison with HAM and the human DM. Although LC can be deemed as a bankable by‐product of cataract surgery, its supply is not unlimited and its biological origins still confer inter and intradonor variability with the theoretical risk of disease transmission. Despite this, the LC possesses ideal optical characteristics with an acceptable thickness, transparency, and resistance to enzymatic degradation.

## CONFLICT OF INTEREST

The authors have declared that there is no conflict of interest.
